# Low levels of soluble TWEAK, indicating on-going inflammation, were associated with depression in type 1 diabetes: a cross-sectional study

**DOI:** 10.1186/s12888-020-02977-3

**Published:** 2020-12-01

**Authors:** Eva O. Melin, Jonatan Dereke, Magnus Hillman

**Affiliations:** 1 Lund University, Faculty of Medicine, Clinical Sciences, Diabetes Research Laboratory, Lund, Sweden; 2 Department of Research and Development, Region Kronoberg, Box 1223, SE-351 12 Växjö, Sweden

**Keywords:** Cardiovascular complications, Continuous subcutaneous insulin infusion, Depression, Galectin-3, HbA1c, High-density lipoprotein-cholesterol, Inflammation, Soluble tumour necrosis factor-like weak inducer of apoptosis, Type 1 diabetes mellitus

## Abstract

**Background:**

Low levels of the soluble tumour necrosis factor-like weak inducer of apoptosis (sTWEAK) and depression are linked to cardiovascular disease. Galectin-3, inadequate glycemic control and low high-density lipoprotein (HDL)-cholesterol levels were previously linked to depression in these patients with type 1 diabetes mellitus (T1DM). The main aim was to explore whether sTWEAK was associated with depression. A secondary aim was to explore diabetes related variables associated with low sTWEAK.

**Methods:**

Cross-sectional design. T1DM patients (*n* = 283, men 56%, age18–59 years) were consecutively recruited from one specialist diabetes clinic. Depression was defined as Hospital Anxiety and Depression Scale-Depression sub scale ≥8 points. Blood samples, anthropometrics and blood pressure were collected, supplemented with data from electronic health records. Enzyme linked immunosorbent assays were used to measure sTWEAK and galectin-3. Low sTWEAK was defined as < 7.2 ng/ml and high galectin-3 as ≥2.6 ng/ml. Multiple logistic regression analyses were performed, calibrated and validated for goodness of fit. We adjusted for age, sex, diabetes duration, galectin-3, metabolic variables, serum-creatinine, smoking, physical inactivity, medication, and cardiovascular complications.

**Results:**

For 29 depressed versus 254 non-depressed patients the prevalence rates were for low sTWEAK: 93 and 68% (*p* = 0.003) and for high galectin-3: 34 and 13% (*p* = 0.005) respectively. HDL-cholesterol levels were lower for the depressed (*p* = 0.015). Patients with low sTWEAK versus high sTWEAK had lower usage of continuous subcutaneous insulin infusion (CSII) (6% versus 17%, *p* = 0.005).

Low sTWEAK (adjusted odds ratio (AOR) 9.0, *p* = 0.006), high galectin-3 (AOR 6.3, *p* = 0.001), HDL-cholesterol (per mmol/l) (AOR 0.1, *p* = 0.006), use of antidepressants (AOR 8.4, *p* < 0.001), and age (per year) (AOR 1.05, *p* = 0.027) were associated with depression.

CSII (AOR 0.3, *p* = 0.003) and depression (AOR 7.1, *p* = 0.009) were associated with low sTWEAK.

**Conclusions:**

Lower levels of sTWEAK and HDL-cholesterol and higher levels of galectin-3 were independently associated with depression in T1DM. These factors might all contribute to the increased risk for cardiovascular disease and mortality previously demonstrated in patients with depression. CSII (inversely) and depression were independently associated with low sTWEAK levels.

## Background

Type 1 diabetes (T1DM) is an autoimmune disease, characterized by insulin deficiency due to pancreatic beta cell loss leading to hyperglycaemia [1]. The introduction of intensive insulin therapy for patients with T1DM has resulted in an increased prevalence of the components of the metabolic syndrome, contributing to an amplified prevalence of cardiovascular complications [2]. Inflammatory disturbances also contribute to cardiovascular disease [3].

In patients with diabetes, depression is associated with increased prevalence of all diabetes-related complications [4], and with increased cardiovascular and all-cause mortality [5]. Depression has been linked to metabolic, autonomic and hypothalamic-pituitary-adrenal (HPA)-axis dysregulations with subsequent disturbances of cortisol secretion [6, 7], and there is growing evidence that immuno-inflammatory changes contribute to the development of depression [6–10]. Insulin deficiency, hyperglycemia as well as episodes of hypoglycemia all have impact on the brain, and might contribute to the development of depression in patients with T1DM [7].

The tumour necrosis factor-like weak inducer of apoptosis (TWEAK) is a transmembrane protein, which is a member of the tumour necrosis factor (TNF)-receptor super family [11]. TWEAK is proteolytically processed by furin which leads to the release of soluble (s)TWEAK [11]. In the reverse process, sTWEAK binds to the functional receptor of TWEAK, Fn14 [12]. The receptor Fn14 is highly upregulated in systemic inflammatory states, which leads to increased sTWEAK binding and subsequently lower levels of sTWEAK [12]. The TWEAK/Fn14 axis plays a beneficial role in tissue repair after acute injury. However, it has been shown that chronic TWEAK/Fn14 axis activation is implicated in the development of cardiovascular disease [12]. High levels of sTWEAK are released by normal arteries, but are diminished in people with chronic vascular damage such as carotid stenosis, coronary artery disease and heart failure [12, 13], resulting in an increased risk for cardiovascular mortality [14]. Proinflammatory effects of TWEAK on astrocytes in vitro implies that TWEAK could play a significant role in brain inflammation [15]. Low sTWEAK levels have been demonstrated in people with T1DM [16], type 2 diabetes mellitus (T2DM) [17], and gestational diabetes [18]. Low sTWEAK levels have also been demonstrated in depressed people without diabetes [19], and in people with bipolar disorder during ongoing manic episodes [20]. Galectin-3 is a beta-galactoside-binding lectin involved in several inflammatory processes [21]. Increased galectin-3 levels have been linked to coronary artery disease [22, 23], heart failure [24, 25], prolonged inflammatory responses in the brain [26], and to cardiovascular and all-cause mortality [25, 27]. Systemic inflammation causes decreased high-density lipoprotein (HDL)-cholesterol levels which contribute both to reduced capacity for reverse cholesterol transport, and to reduced capacity to protect low-density lipoprotein (LDL)-cholesterol from oxidation [28]. Low HDL-cholesterol levels were previously associated with high levels of galectin-3 binding protein in these patients [29]. Inadequate glycemic control contributes to increased cardiovascular and all-cause mortality [30].

Systematic exploration of these T1DM patients showed that self-reported depression was associated with inadequate glycemic control [31], midnight salivary cortisol secretion [32], galectin-3 [33], and inversely with HDL-cholesterol [34]. Depression was not associated with sex, obesity, blood pressure, total cholesterol, LDL-cholesterol, triglycerides, antihypertensive drugs, lipid-lowering drugs, physical inactivity or smoking habits [34]. Neither was depression associated with the inflammatory variables galectin-3 binding protein [29], soluble sCD163 [35], the soluble receptor for advanced glycation end products (sRAGE), nor the extracellular newly identified receptor for advanced glycation end products (EN-RAGE) [36].

We hypothesised that one biological link between depression and cardiovascular complications in depressed patients with T1DM is a chronic inflammatory state due to TWEAK activation with subsequent low levels of sTWEAK. The main aim was to explore whether low levels of sTWEAK were associated with depression in T1DM patients. A secondary aim was to explore diabetes related variables associated with low sTWEAK.

## Methods

### Participants and study design

**Fig. 1:**
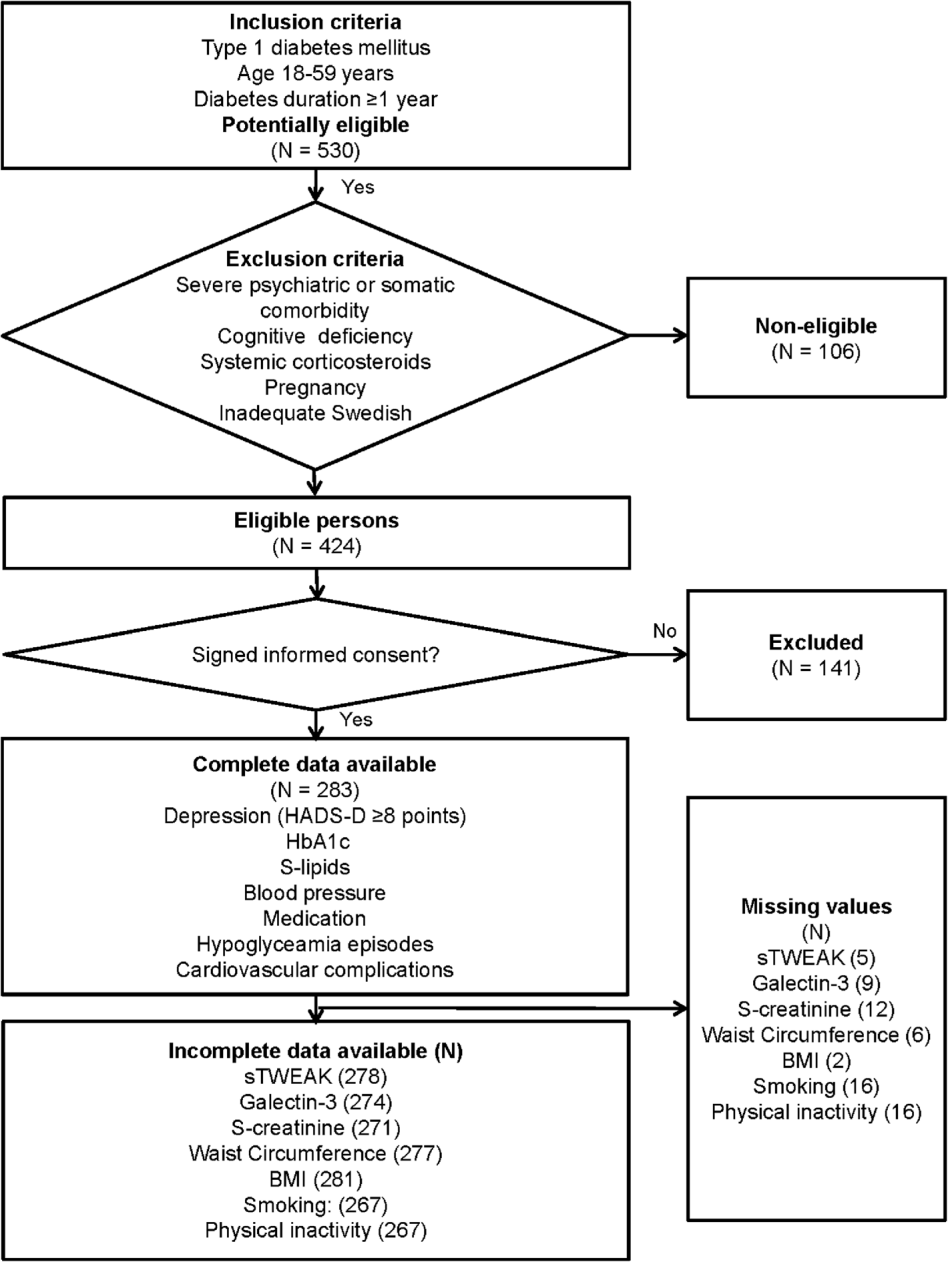
Flow chart describing the included T1DM patients and included variables in this study of depression

The study has a cross sectional design and included 287 patients with T1DM. For inclusion and exclusion criteria, included and missing variables, see Fig. 1. Inclusion criteria were T1DM with ≥1-year duration, in patients 18–59 years of age. Exclusion criteria were pregnancy, severe somatic and psychiatric disorders such as cancer, hepatic failure, end-stage renal disease, Cushing’s disease, severe autoimmune disorders such as systemic lupus erythematosus, psychotic disorders, bipolar disorder, severe personality disorders, severe substance abuse, cognitive deficiency (due to stroke, dementia or mental retardation), or inadequate knowledge of the Swedish language [29, 31–36]. As patients with these disorders were excluded, no specific medications for any of these disorders were used by the included patients. Patients using systemic corticosteroids were excluded [36].

The patients were consecutively recruited from the largest of two specialist diabetes hospital outpatient clinics where all adult patients with T1DM are treated in Region Kronoberg, Sweden [29, 31–36]. The patients who attend the clinic every 6 months for regular follow-up visits were consecutively recruited by specialist diabetes physicians or diabetes nurses during a nine-month period, 25 March 2009 to 28 December 2009 [29, 31–36]. The catchment population was 125,000. A questionnaire was used to assess self-reported depression. Blood samples, anthropometrics and blood pressure were collected, supplemented with data from electronic medical records. Data collection was performed at baseline of a randomized controlled study (RCT) for patients with diabetes, Hemoglobin A1c (HbA1c) > 70 mmol/mol and psychological symptoms (ClinicalTrials.gov: NCT01714986) [37], and the study is one out of several baseline analyses [29, 31–36]. We adjusted for age, sex, diabetes duration, galectin-3, metabolic variables, serum (s)-creatinine, smoking, physical inactivity, medication, and cardiovascular complications.

### Self-reported depression

Depressive symptoms were assessed by the Hospital Anxiety and Depression Scale - the depression subscale (HADS-D), which consists of 7 statements. Each statement has four response alternatives with scores from 0 to 3. The recommended cut-off level was used to define depression: ≥ 8 points as in our previous studies [29, 31–36, 38]. HADS was developed to detect symptoms of depression and anxiety in patients repeatedly searching medical care for somatic complaints, where no somatic disorders were confirmed that could explain their symptoms [38]. No higher levels of emotional awareness are necessary to respond to the statements. A major characteristic of HADS-D is that potential symptoms of somatic disease are not included [38]. According to previous research HADS-D is a useful instrument for detecting symptoms of depression, both at an individual and a collective level, and has been demonstrated to have a good reliability and discriminant validity [39].

### Biochemical analyses

Plasma levels of sTWEAK and galectin-3 were measured using commercially available DuoSet enzyme linked immunosorbent assays (ELISA) kits (R&D Systems, Minneapolis, Mn, USA) and optimised for human plasma. The analyses were run according to the manufacturer’s instructions. The samples were diluted 1:5 and 1:2, and the intra-assay coefficients of variation were 1.8 and 4.3% respectively for sTWEAK and galectin-3. All samples were run as duplicates. High galectin-3 levels were defined as ≥2.6 ng/ml as in our previous research [33].

HbA1c and serum-lipids were collected after an overnight fast and they were analysed with an Olympus automated clinical chemistry analyser (Olympus AU®, Tokyo, Japan). (corresponding to the 72nd percentile) as in our previous research [31]. High HbA1c levels were defined as > 70 mmol/mol (NGSP > 8.6%). The intra-assay coefficients of variation were for HbA1c < 1.2%; total cholesterol < 2.1%; HDL-cholesterol < 3.0%; LDL-cholesterol < 2,6%; and for triglycerides < 2.2%.

S-creatinine was assayed by an AU2700® instrument (Beckman Coulter, Brea, Ca, USA). The intra-assay coefficient of variation was < 3%.

### Anthropometrics and blood pressure

Waist circumference (WC), weight, length and blood pressure were measured according to standard procedures by a nurse. Body Mass Index (BMI) (kg/m^2^) was calculated. Abdominal obesity was defined as WC ≥ 1.02 m for men and as WC ≥ 0.88 m for women as in our previous studies [29, 31–36, 40].

### Insulin resistance

A ratio between triglycerides and HDL-cholesterol was calculated for the estimation of insulin resistance [41].

### Episodes of hypoglycemia

A severe episode of hypoglycemia was defined as hypoglycemia to such a degree that the patient needed help from another person. Episodes during the last 6 months prior to recruitment were registered [29, 31–36].

### Smoking and physical inactivity

Smokers were defined as having smoked any amount of tobacco during the last year. Physical activity was dichotomized into physical inactivity which was defined as less than 30 min of moderate activities once a week, and physical activity which represents all other levels of physical activity [29, 31–36].

### Cardiovascular complications

Cardiovascular complications were defined as ischemic heart disease, cardiac failure, stroke, or transient ischemic attack [29, 31–36].

### Diabetes medication

Patients used either multiple daily insulin injections (MDII) or continuous subcutaneous insulin infusion (CSII) [29, 31–36].

### Antidepressants

Antidepressants were SSRIs (ATC codes N06AB04 or N06AB10); SNRIs (ATC code N06AX16); combined serotonin and norepinephrine reuptake inhibitors (ATC code N06AX21); tricyclic antidepressants (ATC code N06AA04); and/or tetracyclic antidepressants (ATC code N06AX11). The use of antidepressants was dichotomized into users and non-users [29, 31–36].

### Lipid-lowering drugs and indications for treatment of hyperlipidemia

Lipid-lowering drugs were hydroxy-methylglutaryl coenzyme A (HMG-CoA)-reductase inhibitors (statins), (ATC-code C10AA). The use of lipid-lowering drugs was dichotomized into users and non-users of lipid-lowering drugs [29, 31–36].

Indications for lipid-lowering drugs were TC > 4.5 mmol/l (> 1.74 mg/dl) and/or LDL-cholesterol > 2.5 mmol/l (> 97 mg/dl) according to the Swedish national guidelines in 2009 [42].

### Antihypertensive drugs and indications for treatment of hypertension

Antihypertensive drugs included calcium antagonists (ATC codes C08CA01–02); angiotensin-converting enzyme (ACE) inhibitors (ATC codes C09AA-BA); angiotensin II antagonists (ATC codes C09CA-DA); diuretics (ATC codes C03AA03 or C03CA01); and/or selective beta-adrenoreceptor antagonists (ATC code C07AB). The use of antihypertensive drugs was dichotomized into users and non-users of antihypertensive drugs [29, 31–36].

Indications for antihypertensive drugs were systolic blood pressure > 130 mmHg and/or diastolic blood pressure > 80 mmHg according to the Swedish national guidelines in 2009 [42].

### Statistical analysis

Analysis of data distribution using histograms revealed that age, diabetes duration, sTWEAK, galectin-3, triglycerides, systolic and diastolic blood pressure, were not normally distributed. Data were presented as median (quartile (q)_1_, q_3_), and analyses were performed with Mann-Whitney *U* test. Fisher’s Exact Test (two-tailed) was used to analyse categorical data, and data were presented as N (%). The 60th, 65th, 70th and 75th percentiles of sTWEAK and log-transformed sTWEAK (Lg10) were tried against depression in a backward elimination multiple logistic regression analysis, and the percentile with the highest association was defined as low sTWEAK and was therefore used in the further analyses. Crude odds ratios (CORs) for the associations with depression and with low sTWEAK (< 7.2 ng/ml) were calculated for all included variables. Variables with *p* < 0.10 for the CORs were entered into multiple logistic regression analyses (Backward: Wald) with depression and low sTWEAK as dependent variables [43]. The Hosmer and Lemeshow test for goodness-of-fit and Nagelkerke R^2^ were used to evaluate each multiple logistic regression analysis model. Confidence intervals (CIs) of 95% were used. *P* < 0.05 was considered statistically significant. SPSS® version 25 (IBM, Chicago, Il, USA) was used.

## Results

In this study 283 patients with T1DM (56% men), 18–59 years old, diabetes duration 1–55 years were included. Baseline characteristics and laboratory results are compared between 29 patients with depression and 254 patients without depression in Table 1. All patients used either MDII (91%) or CSII (9%). The depressed patients had lower median sTWEAK (*p* = 0.017) and median cholesterol (*p* = 0.044); and had higher prevalence of high galectin-3 (*p* = 0.005), high HbA1c (*p* = 0.026), use of antidepressants (*p* < 0.001), and cardiovascular complications (*p* = 0.012).

**Table 1:** Baseline characteristics, laboratory results, and comparisons between 29 depressed and 254 non-depressed patients with T1DM

		Depression		
	All	Yes	No	***P*** - value ^***a***^
*N*	283	29	254	
Age (years)	(18–59)	49 (38, 53)	42 (31, 50)	0.036
Diabetes duration (years)	(1–55)	21 (11, 35)	20 (10, 29)	0.45
Sex				
Women	124 (44)	13 (45)	111 (44)	> 0.99 ^b^
Men	159 (56)	16 (55)	143 (56)	
Depression	29 (10)	–	–	–
sTWEAK (ng/ml) ^c^	(0.5–1618)	1.5 (1.2, 2.9)	2.7 (1.3, 13.4)	0.017
Galectin-3 (ng/ml) ^d^	(0.001–100.0)	1.3 (0.8, 3.0)	0.9 (0.5, 1.6)	0.011
High galectin-3 (≥2.6 ng/ml) ^d^	41 (15)	10 (34)	31 (13)	0.005 ^b^
HbA1c				
mmol/mol	(25–110)	68 (55, 73)	63 (54, 70)	0.056
%	(4.4–12.2)	8.4 (7.2, 8.8)	7.9 (7.1, 8.5)	
High HbA1c (> 70 mmol/mol (> 8.6%))	75 (26)	13 (45)	62 (24)	0.026 ^b^
Total cholesterol (mmol/l)	(2.1–10.9)	4.3 (4.0, 4.8)	4.6 (4.1, 5.2)	0.044
LDL-cholesterol (mmol/l)	(0.6–8.3)	2.8 (2.2, 3.2)	2.8 (2.4, 3.3)	0.37
Triglycerides (mmol/l)	(0.06–5.9)	0.9 (0.7, 1.5)	0.9 (0.7, 1.2)	0.35
HDL-cholesterol (mmol/l)	(0.3–2.7)	1.3 (1.2, 1.6)	1.6 (1.3, 1.8)	0.015
Triglycerides/HDL-cholesterol ratio	(0.04–5.9)	0.6 (0.5–1.4)	0.6 (0.4–0.9)	0.23
S-Creatinine (μmol/l) ^e^	(28–182)	72 (63, 80)	70 (62, 78)	0.94
Abdominal obesity ^f^	46 (17)	6 (21)	40 (16)	0.43 ^b^
BMI (kg/m^2^) ^g^	(17.8–45.2)	23.8 (21.8, 27.3)	24.8 (22.8, 27.5)	0.21
Systolic BP (mm Hg)	(90–160)	120 (112, 135)	120 (110, 130)	0.99
Diastolic BP (mm Hg)	(55–100)	70 (68, 78)	70 (70, 75)	0.90
Hypoglycemia (severe episodes)	12 (4)	2 (7)	10 (4)	0.36 ^b^
Smoking ^h^	28 (10)	5 (18)	23 (10)	0.19 ^b^
Physical inactivity ^i^	29 (11)	5 (18)	24 (10)	0.20 ^b^
Continuous subcutaneous insulin infusion	25 (9)	3 (10)	22 (9)	0.73 ^b^
Antidepressants	22 (8)	9 (31)	13 (5)	< 0.001 ^b^
Lipid lowering drugs	130 (46)	14 (48)	116 (46)	> 0.84 ^b^
Antihypertensive drugs	89 (31)	10 (34)	79 (31)	0.68 ^b^
Cardiovascular complications	10 (4)	4 (14)	6 (2)	0.012 ^b^

Data are presented as (min-max), median (q_1_, q_3_), or N (%). ^a^ Mann-Whitney U Test unless otherwise indicated

^b^ Fisher’s Exact Test. Missing values (N): ^c^ 5; ^d^ 9; ^e^ 12; ^f^ 6 ^g,^ 2; ^h, i^ 16

Associations with depression are presented for log transformed sTWEAK and four levels of low sTWEAK, representing the 60th, 65th, 70th, and 75th percentiles of sTWEAK, are presented in Table 2. sTWEAK < 7.2 ng/ml (< 70th percentile) showed the highest association with depression (adjusted odds ratio (AOR) 6.5, *p* = 0.010) compared to the 60th, 65th, 75th percentiles and log transformed sTWEAK (all *p*-values > 0.48 for the AORs).

**Table 2:** Elimination analyses between log transformed sTWEAK, 4 levels of dichotomized sTWEAK and depression

		Depression						
	N (%)	Yes (Median (q_1_, q_3_) or N (%))	No (Median (q_1_, q_3_) or N (%))	***P*** - value ^***a***^	COR (95% CI)	***P*** - value ^***b***^	AOR (95% CI)	***P*** - value ^***c***^
sTWEAK (Lg10)	–	0.2 (0.1–0.5)	0.4 (0.1–1.1)	0.017 ^d^	0.4 (0.2–0.9)	0.027	1.7 (0.4–7.6)	0.48
sTWEAK < 3.9 (ng/ml)	167 (60)	24 (83)	143 (57)	0.009	3.6 (1.3–9.6)	0.012	1.4 (0.4–5.0)	0.61
sTWEAK < 6.1 (ng/ml)	181 (65)	25 (86)	156 (63)	0.013	3.7 (1.3–11.0)	0.018	0.5 (0.04–6.2	0.58
sTWEAK < 7.2 (ng/ml)	195 (70)	27 (93)	168 (68)	0.003	6.5 (1.5–28.0)	0.012	6.5 (1.5–28.0)	0.012
sTWEAK < 11.9 (ng/ml)	209 (75)	27 (93)	182 (73)	0.021	5.0 (1.2–21.5)	0.032	0.0 (0.0-)	> 0.99

*N* = 278. ^a^ Fisher’s Exact Test unless indicated. ^b^ Logistic regression analysis (simple)

^c^ Multiple logistic regression analysis (Backward: Wald). ^d^ Mann-Whitney U test

In Table 3 associations with depression are presented. Age (per year) (AOR 1.05, *p* = 0.027), low sTWEAK (< 7.2 ng/ml) (AOR 9.0, *p* = 0.006), high galectin-3 (AOR 6.3, *p* = 0.001), HDL-cholesterol (per mmol/l) (inversely) (AOR 0.1, *p* = 0.006), and use of antidepressants (AOR 8.4, *p* < 0.001), were associated with depression.

**Table 3:** Variables associated with depression in patients with T1DM

	Depression			
	COR (95% CI)	***P*** - value	AOR (95% CI)	***P*** - value
Age (per year)	1.04 (1.00–1.07)	0.046	1.05 (1.01–1.10)	0.027
Diabetes duration (per year)	1.01 (0.98–1.04)	0.46	–	–
Sex (women)	1.0 (0.5–2.3)	0.91	–	–
Low sTWEAK (< 7.2 ng/ml)	6.5 (1.5–28.0)	0.012	9.0 (1.9–43.2)	0.006
High galectin-3 (≥2.6 ng/ml)	3.6 (1.5–8.5)	0.003	6.3 (2.2–17.8)	0.001
High HbA1c (> 70 mmol/mol (> 8.6%))	2.5 (1.1–5.5)	0.021	2.4 (0.9–6.3)	0.067
Total cholesterol (per mmol/l)	0.6 (0.4–1.0)	0.040	0.7 (0.4–1.2)	0.19
LDL-cholesterol (per mmol/l)	0.7 (0.4–1.2)	0.23	–	–
Triglycerides (per mmol/l)	1.3 (0.8–2.0)	0.23	–	–
HDL-cholesterol (per mmol/l)	0.3 (0.1–0.8)	0.020	0.1 (0.03–0.6)	0.006
Triglyceride/HDL-cholesterol ratio (per unit)	1.5 (1.0–2.3)	0.038	1.2 (0.6–2.4)	0.56
S-Creatinine (per μmol/l)	1.00 (0.98–1.02)	0.72	–	–
Abdominal obesity	1.4 (0.5–3.7)	0.47	–	–
BMI (per kg/m^2^)	1.0 (0.9–1.1)	0.38	–	–
Systolic BP (per mm Hg)	1.00 (0.96–1.03)	0.90	–	–
Diastolic BP (per mm Hg)	1.00 (0.95–1.06)	0.98	–	–
Hypoglycemia (severe episodes)	1.8 (0.4–8.6)	0.46	–	–
Smoking	2.0 (0.7–5.9)	0.19	–	–
Physical inactivity	1.9 (0.7–5.6)	0.22	–	–
Continuous subcutaneous insulin infusion	1.2 (0.3–4.3)	0.76	–	–
Antidepressants	8.3 (3.2–21.9)	< 0.001	8.4 (2.7–26.1)	< 0.001
Lipid lowering drugs	1.1 (0.5–2.4)	0.79	–	–
Antihypertensive drugs	1.2 (0.5–2.6)	0.71	–	–
Cardiovascular complications	6.6 (1.7–25.0)	0.005	1.1 (0.2–6.3)	0.92

*N* = 273; Multiple logistic regression analyses (Backward: Wald); Nagelkerke R Square 0.340; Hosmer and Lemeshow test 0.549

Comparisons between patients with low sTWEAK levels (< 7.2 ng/ml), and high sTWEAK levels (≥ 7.2 ng/ml) are presented in Table 4. Fourteen percent of the patients with low sTWEAK were depressed and 2% of the patients with high sTWEAK were depressed (*p* = 0.003). Patients with low sTWEAK used CSII (6%) to a lower extent than patients with high sTWEAK (17%) (*p* = 0.005).

**Table 4:** Comparisons between 195 T1DM patients with low and 83 T1DM patients with high levels of sTWEAK

	sTWEAK		
	Low levels (< 7.2 ng/ml)	High levels (≥7.2 ng/ml)	***P - value*** ^***a***^
*N*	195	83	
Age (years)	42 (32, 51)	42 (29, 51)	0.79
Diabetes duration (years)	20 (11, 30)	18 (9, 29)	0.58
Sex			
Women	89 (46)	30 (36)	0.15 ^b^
Men	106 (54)	53 (64)	
Depression	27 (14)	2 (2)	0.003 ^b^
High galectin-3 (≥2.6 ng/ml) ^c^	27 (14)	14 (17)	0.58 ^b^
High HbA1c (> 70 mmol/mol (> 8.6%))	53 (27)	22 (26)	> 0.99 ^b^
Total cholesterol (mmol/l)	4.6 (4.1, 5.2)	4.5 (4.0, 5.0)	0.13
LDL (mmol/l)	2.9 (2.4, 3.4)	2.8 (2.4, 3.2)	0.34
Triglycerides (mmol/l)	0.9 (0.7, 1.3)	0.8 (0.7, 1.2)	0.19
HDL (mmol/l)	1.5 (1.3, 1.8)	1.6 (1.3, 1.8)	0.71
Triglycerides/HDL-cholesterol ratio	0.6 (0.4–0.9)	0.6 (0.4–0.8)	0.23
S-Creatinine (μmol/l) ^d^	70 (62, 77)	70 (62, 80)	0.44
Abdominal obesity ^e^	33 (17)	11 (13)	0.48 ^b^
BMI (kg/m^2^) ^f^	24.6 (22.4, 27.9)	24.6 (23.1, 26.1)	0.92
Systolic BP (mm Hg)	120 (110, 130)	120 (110, 130)	0.34
Diastolic BP (mm Hg)	70 (70, 75)	70 (65, 78)	0.54
Hypoglycemia (severe episodes)	7 (4)	4 (5)	0.74 ^b^
Smoking ^g^	20 (11)	8 (10)	> 0.99 ^b^
Physical inactivity ^h^	24 (13)	5 (6)	0.19 ^b^
Continuous subcutaneous insulin infusion	11 (6)	14 (17)	0.005 ^b^
Antidepressants	16 (8)	6 (7)	> 0.99 ^b^
Lipid lowering drugs	92 (47)	36 (43)	0.60 ^b^
Antihypertensive drugs	62 (32)	26 (31)	> 0.99 ^b^
Cardiovascular complications	8 (4)	2 (2)	0.73 ^b^

*N* = 278. Data are presented as median (q_1_, q_3_) or N (%). ^a^ Mann-Whitney U test unless otherwise indicated

^b^ Fisher’s Exact Test. Missing values: ^c^ 4; ^d^ 12; ^e^ 6; ^f^ 2; ^g, h^ 16

Associations with low sTWEAK levels are presented in Table 5. Depression (AOR 7.1, *p* = 0.009) and the use of CSII (inversely) (AOR 0.3, *p* = 0.003) were independently associated with low sTWEAK (< 7.2 ng/ml).

**Table 5:** Variables associated with low sTWEAK levels

	Low sTWEAK (< 7.2 ng/ml)			
	COR (95% CI)	***P - value***	AOR (95% CI)	***P - value***
Age (per year)	1.00 (0.98–1.03)	0.73	–	–
Diabetes duration (per year)	1.00 (0.98–1.02)	0.71	–	–
Sex (women)	1.5 (0.9–2.5)	0.14	–	–
Depression	6.5 (1.5–28.0)	0.012	7.1 (1.6–31.4)	0.009
High galectin-3 (≥2.6 ng/ml)	0.8 (0.4–1.6)	0.49	–	–
High HbA1c (> 70 mmol/mol (> 8.6%))	1.0 (0.6–1.8)	0.91	–	–
Total cholesterol (mmol/l)	1.2 (0.9–1.6)	0.19	–	–
LDL (mmol/l)	1.2 (0.9–1.7)	0.29	–	–
Triglycerides (mmol/l)	1.3 (0.9–2.0)	0.18	–	–
HDL (mmol/l)	0.8 (0.4–1.7)	0.60	–	–
Triglycerides/HDL-cholesterol ratio	1.5 (0.9–2.6)	0.11	–	–
S-Creatinine (μmol/l)	1.00 (0.98–1.01)	0.63	–	–
Abdominal obesity	1.4 (0.6–2.8)	0.42	–	–
BMI (per kg/m^2^)	0.7 (0.4–1.3)	0.26	–	–
Systolic BP (mm Hg)	1.01 (0.99–1.03)	0.33	–	–
Diastolic BP (mm Hg)	1.00 (0.96–1.03)	0.63	–	–
Hypoglycemia (severe episodes)	0.7 (0.2–2.6)	0.64	–	–
Smoking	1.0 (0.4–2.5)	0.92	–	–
Physical inactivity	2.1 (0.8–5.9)	0.14	–	–
Continuous subcutaneous insulin infusion	0.3 (0.1–0.7)	0.004	0.3 (0.1–0.6)	0.003
Antidepressants users	1.1 (0.4–3.0)	0.78	–	–
Lipid lowering drugs	1.2 (0.7–2.0)	0.56	–	–
Antihypertensive drugs	1.0 (0.6–1.8)	0.94	–	–
Cardiovascular complications	1.7 (0.4–8.3)	0.49	–	–

*N* = 278; Multiple logistic regression analyses (Backward: Wald); Nagelkerke R Square: 0.094; Hosmer and Lemeshow test 0.802

## Discussion

The main finding of this study of adult patients with T1DM was that the depressed patients had lower levels of sTWEAK than the non-depressed patients. Lower levels of sTWEAK and HDL-cholesterol, higher levels of galectin-3, the use of antidepressants, and age were independently associated with depression. The depressed patients had also higher levels of HbA1c, but the association between high HbA1c and depression was not independent in this context. The use of CSII was inversely associated with low sTWEAK.

Depression is a serious disease with somatic implications, including cardiovascular disease and all-cause mortality [4–7]. Three independent risk factors or risk markers for cardiovascular disease were demonstrated in these depressed patients with T1DM. Low sTWEAK levels [12–14] and high galectin-3 levels [22–25, 27] have previously been linked to the development of cardiovascular disease and increased mortality. HDL-cholesterol levels decrease in inflammatory states [28, 29]. HDL-cholesterol is an established risk marker for cardiovascular disease, but the role of HDL-cholesterol as a causal factor in cardiovascular disease is disputed [44]. In previous research, we have not demonstrated any higher prevalence of metabolic disturbances in the depressed than in the non-depressed, except for increased HbA1c and lower HDL-cholesterol in the depressed patients [31, 34]. In this study we added a proxy for insulin resistance [41], which was not associated with depression. Increased HbA1c levels without associated obesity or signs of insulin resistance in the depressed patients could be due to inadequate insulin supply. Insulin deficiency has been suggested as one reason for the development of depression in patients with T1DM [7, 45]. Users of MDII compared to users of CSII had more often low levels of sTWEAK. We haven’t found any previous research exploring the associations between sTWEAK and CSII.

One difficulty we had to address was that there is no established consensus regarding normal sTWEAK levels. Therefore, we explored and compared the associations between log transformed sTWEAK, four different definitions of low sTWEAK levels and depression. Low sTWEAK levels, defined as levels below the 70th percentile (< 7.2 ng/ml), showed the highest association with depression, and this cut-off level was therefore chosen in the further analyses.

To our knowledge, a potential association between sTWEAK and depression hasn’t previously been explored in patients with T1DM. Neither has it been explored whether low sTWEAK, high galectin-3, high HbA1c and low HDL-cholesterol were independently associated with depression. We only found one previous study exploring the association between sTWEAK and depression, and that study was performed in a population without diabetes [19]. Their findings of an association between low sTWEAK levels and depression is in accordance with our findings. Another study showed that manic episodes were linked to low levels of sTWEAK levels [20]. Both depressive states and manic episodes may be symptoms of brain inflammation. Our findings of low sTWEAK levels in the depressed patients imply activation of TWEAK, which has proinflammatory effects on the astrocytes in the brain, which have been demonstrated in vitro [15]. According to previous research, galectin-3 may contribute to microglia activation with sustained inflammatory responses in the brain [26]. These findings are potentially very important as it has been demonstrated in previous research that both astrocytes and microglia are involved in the development of depression [46, 47].

There are several subjects for further research. As this is a cross-sectional study, we can’t clarify whether the depressed state leads to immunological disturbances, or if these immunological disturbances lead to a depressed state. To answer this question, it will be necessary to perform longitudinal studies. We will perform a follow-up exploring the impact of sTWEAK and galectin-3 on cardiovascular complications, comparing with conventional diabetes related risk factors. According to previous research, some antidepressants attenuate immuno-inflammatory changes [9]. Whether antidepressants may have impact on the levels of sTWEAK and galectin-3 is a subject for further exploration. As immuno-inflammatory changes and the activation of the hypothalamic pituitary axis are part of the stress response, which is involved in depression, it would also be interesting to explore associations between sTWEAK, galectin-3 and cortisol secretion [9]. To compare TWEAK levels between users of MDII and users of CSII in a future larger study would be very interesting. If our findings would be confirmed in a larger study, it would be a very important finding supporting the choice of CSII over MDII. Finally, development of novel therapeutics against the TWEAK/Fn14 axis may be of value both for the treatment or prevention of depression and cardiovascular disease.

Strengths of the study are that inclusion and exclusion criteria were well defined. No patients using systemic corticosteroids, or specific drugs for psychotic or bipolar disorders were included. The findings of this study are new as exploration of the association between sTWEAK and depression has not previously been performed in a clinically well-defined setting of T1DM. Neither has the mode of insulin distribution been explored against sTWEAK. The results were controlled for relevant variables which in previous research have been linked to either depression or cardiovascular complications, or to both. The logistic regression models were elaborated for the associations, and calibrated and validated for goodness of fit with the data variables. Precise ELISA techniques were used, and the assays showed low intra-assay coefficients of variation for both sTWEAK and galectin-3. One weakness was that there was no control group with persons without diabetes. Another weakness was that there were very few patients with cardiovascular complications, so no further explorations of associations with cardiovascular complications could be performed. A third weakness was that depression was not assessed by a clinical interview. However, the association between the use of antidepressants and self-reported depression in this study was high, indicating that depression assessed by HADS-D had clinical significance.

## Conclusions

The hypothesis that the depressed patients with T1DM had lower levels of sTWEAK than the non-depressed was confirmed. Low levels of sTWEAK and HDL-cholesterol and high levels of galectin-3 were independently associated with depression in T1DM. These disturbances have previously been associated with cardiovascular disease and mortality, and might contribute to the increased risk for cardiovascular disease and mortality previously demonstrated in T1DM patients with depression. We also found that the users of CSII had lower prevalence of low sTWEAK levels than the users of MDII.

## Data Availability

All data are saved at SPSS files for at least 15 years at the Department for Research and Development, Region Kronoberg, Växjö, Sweden. The data sets are not publicly available as individual privacy could be compromised. The data set is available from the corresponding author upon reasonable request.

## Abbreviations

AOR: Adjusted Odds Ratio
BMI: Body Mass Index
CI: Confidence interval
COR: Crude Odds Ratio
CSII: Continuous subcutaneous insulin infusion
HADS-D: Hospital Anxiety and Depression-Depression subscale
MDII: Multiple daily insulin injections
sTWEAK: Soluble tumour necrosis factor-like weak inducer of apoptosis
T1DM: Type 1 diabetes mellitus
T2DM: Type 2 diabetes mellitus
TNF-receptor: tumour necrosis factor-receptor
WC: Waist circumference
